# Supplementary material for: Are carbon emissions trading and green financial instruments synergistic? -Comprehensive quantitative research based on content analysis
Source: PLoS One. 2024 Mar 7;19(3):e0298601. doi: 10.1371/journal.pone.0298601 (PMC10919601; doi:10.1371/journal.pone.0298601)
Supplement: S1 Data — (ZIP) [file pone.0298601.s001.zip › Data/地级市Z_F工作报告.zip!/║■─╧/╔█╤⌠╩╨/└┤╫╘╣1⁄2╓┌║┼í╛┬φ┐╦╩2╛▌═°í┐_2013.docx]

各位代表：

  现在，我代表市人民政府，向大会报告工作，请予审查。请各位政协委员和列席人员提出意见。

  一、五年来的工作回顾

  五年来，在省委、省政府及市委的坚强领导下，在市人大、市政协的监督和支持下，我们团结带领全市各族人民，开拓进取，扎实工作，在金融危机冲击、自然灾害多发等情况下，克服制约发展的诸多困难，超额完成了市十四届人大会议确定的目标任务。

  1、综合实力跃上大台阶。这五年是我市历史上发展速度最快、发展质量最好的时期之一。主要经济指标成倍增长，大多实现翻番，多数进入全省“第二方阵”。地区生产总值连续跨越六个台阶，2012年突破1000亿元大关，达到1050亿元（预计数，下同），增长12.5%，是2007年的2.3倍，年均增长12.8%。财政收入85亿元，增长28%，是2007年的2.8倍，年均增长23%；其中市本级财政收入21.6亿元，年均增长28.3%。金融机构存款余额1325亿元、贷款余额532亿元，分别是2007年末的2.4倍、2.6倍，年均增长18.8%、20.7%。2012年新增贷款110亿元，增量存贷比达到52%。三次产业比例由2007年的25.5:33.2:41.3调整为24.2:39.3:36.5。2012年规模工业企业达到760家，其中产值过亿元企业360家，比2007年分别增加158家和315家。规模工业增加值345亿元，增长16%，是2007年的4倍，年均增长21.2%。其中建材、食品和机械三大产业产值突破100亿元，分别为170亿元、165亿元和135亿元，年均增长48%、46.1%和58.6%。全市省级工业园区和工业集中区12个，园区工业增加值180亿元，占全部规模工业增加值的52%。农林牧渔增加值254亿元，年均增长5.2%。粮食总产连续五年稳定在340万吨以上，储备总量稳步增加。生猪、肉牛出栏数和奶牛存栏数均居全省首位，养殖业总产量名列全省前茅。森林覆盖率57.56%，比2007年提高2.06个百分点。农产品加工规模企业162家，其中国家级龙头企业2家、省级龙头企业31家。农业综合开发投资23.4亿元，完成土地治理项目78个，改造中低产田51.3万亩，改良草山4.1万亩，建设高标准农田13.1万亩。全力推进12个示范镇、32个示范片和800个示范村的办点示范，新农村建设成效显著。农业综合机械化水平48%，比2007年提高9个百分点。2012年第三产业增加值、社会消费品零售总额、旅游总收入分别为378亿元、381亿元、103亿元，增长14.4%、16%、31%。我市成功列入全国农产品现代流通综合试点城市，发制品获批全国外贸转型升级示范基地。首家按五星级标准建设的华天大酒店正式营业。新改造标准化农贸市场18个，建成标准化农家店4056家。崀山申世遗成功，成为全国第八处、全省第二处世界自然遗产。全市已拥有国家A级旅游景区10处、国家森林公园4个、国家地质公园1个、国家级自然保护区2处、国家级风景名胜区4个。

  2、争取重大政策和重点项目前所未有。这五年是我市争取重大政策和重点项目最多、城乡面貌变化最大的时期之一。先后成功纳入大湘西开发、国家武陵山区域发展与集中连片扶贫攻坚、全国问题型老工业基地调整改造、全国资源型城市可持续发展规划等重大政策扶持范围。累计完成固定资产投资2763.5亿元，年均增长35.3%，2012年完成767亿元，增长36.5%。建成邵怀、邵衡、邵永、娄新4条高速公路，总里程达214公里，在建洞新、邵安、邵坪、怀通4条高速公路210公里。完成国省干线公路改造和大中修1100公里。新建农村公路16994.5公里，其中通乡公路1372.6公里，通达工程1331.4公里、通畅工程14290.5公里，实现100%的行政村通公路、100%的乡镇和90%的行政村通水泥路（油路）。9个县市和北塔区均建有二级以上客运车站，建成78个农村客运站和546个招呼站。改造危桥108座，渡改桥68座。娄邵铁路扩改和沪昆高速铁路建设进展顺利。怀邵衡铁路和武冈机场进入立项审批，邵东机场改建前期工作已经启动。备受社会关注的邵新公路建成通车。资江干流航道和码头得到疏浚和修复。我市被评为全国农村公路建设示范市、全国农村公路建设质量监管先进市、全国交通运输行业文明创建工作先进市，2008年确定为全国公路交通枢纽城市。除险加固了1座大型水库、15座中型水库、341座小型水库，有6个县列入全国小型农田水利建设重点县。27个中小河流治理项目全面启动。我市整体纳入衡邵干旱走廊综合治理范围。犬木塘水库、市区第二水源建设项目列为全省重点项目。新增装机165万千瓦，新增发电量13.6亿千瓦时。宝庆电厂一期、南山风电一期建成发电，筱溪、晒谷滩等电站并网发电，隆回县生物质电厂竣工。全市83%的村完成农网改造。建成沼气池61131口、养殖小区和联户沼气工程867处、大中型沼气工程13处。城市基础设施建设“百亿投资计划”顺利实施，市区建成区面积扩大到57平方公里。魏源东路、西湖南路、雪峰南路、大祥路、财神路等竣工通车，魏源广场、双清公园、水府游园改造完工，蔡锷广场、南江嘴公园、时代公园建成开园，东互通大道、学院路等扩改工程进展顺利。人防1505指挥所建成使用。自来水过江工程和管输天然气入邵工程竣工。新开公交线路4条，更新公交车259台。建成11个城镇污水处理厂、9个生活垃圾无害化填埋场和1个医疗垃圾处置中心，中心城镇污水处理率78.36%，生活垃圾和医疗垃圾集中处理率分别为75%和80%。市区成功创建省级卫生城市。县城和中心城镇建设步伐不断加快。新宁连续两届成功创建省级文明县城，洞口、邵东、武冈、隆回成功创建省级卫生城市。全市城镇化率36%，比2007年提高7个百分点。

  3、改革开放取得重大突破。这五年是我市改革力度最大、招商成效最好、利用外资最多的时期之一。完成企业改制110户，安置职工2.2万人，累计盘活企业资产12亿元。2012年启动36户国企改制，有19户已通过改制方案。在全省率先完成文化体制转企改制。集体林权制度改革任务主体完成。大圳改革改制强力推进。殡葬管理改革成效明显。公路管理、财税金融、医药卫生、粮食流通、国有林场、社保医保、投融资体制、事业单位体制和资源环境价格等各项改革协同推进。农村金融体制改革成绩突出，组建2家农村商业银行、1家村镇银行和6家小额贷款公司。直接融资较有成效，发行城投债募集22亿元。13家企业进入省重点上市后备资源库，维克液压完成上市申报。五年累计引进外资项目101个，实际利用外资5.6亿美元，年均增长15.9%。18家世界500强企业、20家中国500强企业落户我市。2012年成功举办规格高、规模大、效果好的邵商大会，签订招商合同资金508亿元。五年累计完成进出口总额16亿美元，年均增长22.4%。境外投资企业142家，占全省的70%，实现营业额44.4亿美元，对外劳务输出2.8万人，年均分别增长73.6%、73.8%和16.6%。株洲出入境检验检疫局邵阳工作组正式挂牌，韶山海关驻邵机构正在筹建。

  4、社会事业全面进步。这五年是我市各项社会事业发展最快、社会公共服务质量最好的时期之一。投入资金18.2亿元，完成64所农村初中校舍改造、243个薄弱学校改造、29所边远农村学校的教师周转宿舍改建和11个学前教育中心建设。建成义务教育合格学校540所、寄宿制学校486所。义务教育“三率”全部达标。基本普及高中阶段教育。职业教育特色鲜明。民办教育充满活力。高等教育规模壮大，邵阳学院申硕成功。获批省级工程技术研究中心3个，市级工程技术研究中心12个。获省科技进步三等奖以上项目15项。申请专利3628件，授权2314件。高新技术产业总产值达到450亿元，是2007年的10倍。我市列入“全国知识产权试点城市”。免费送戏4800场，送电影32万多场。获省级以上艺术创作奖项120多项，其中戏剧类最高奖项“梅花奖”2项。新建和改建乡镇综合文化站191个，建成农家书屋5542个。绥宁寨市获评国家级历史文化名镇。国家级非物质文化遗产11项，国家级重点文物保护单位5处。成功举办两届市文化艺术节。广播电视“村村通”工程扎实推进，市区数字电视整体转换率100%。新增体育场地面积80万平方米，开展体育活动550项次，获省级以上赛事金牌130多枚。5个单位获国家“群众体育先进单位”称号。城乡卫生服务网络不断健全，医疗和公共卫生服务能力明显增强。新农合覆盖全体农民。基本药物制度全面推行。免费实施免疫规划、健康建档、白内障复明、慢病管理等11类基本公共卫生服务项目和6类重大公共卫生服务项目。出生人口素质显著提高，人口自然增长率保持在6.5‰左右。实施土地开发项目317个，新增耕地5.36万亩，连续12年实现耕地占补平衡。矿产开发秩序进一步规范，煤矿由162家整合为110家。环境综合整治力度逐年加大，关闭规模小、污染重企业262家，完成工程减排项目263个，超额完成节能减排任务。饮用水水源水质全部达到国家Ⅲ类标准，城区空气质量达到国家二级标准。新宁、绥宁、城步、隆回4县成为“全国生态示范区”。

  5、城乡群众得到最多实惠。这五年是我市解决民生问题力度最大、人民群众生活水平提升最快的时期之一。城镇居民人均可支配收入和农民人均纯收入分别为15610元和5160元，年均增长11.7%和13.2%。五年新增农村劳动力转移就业44.7万人次、城镇就业23.7万人次，完成农村劳动力转移培训27.2万人次、创业培训1.8万人次、扶贫培训85万人次，城镇登记失业率控制在4.5%以内。城镇养老、机关养老、失业、工伤、城镇职工医保、城镇居民医保和生育保险参保人数分别为40.2万人、15.4万人、27.5万人、46.8万人、54.3万人、88.8万人和34.4万人。农村养老保险参保率99.3%。累计发放社会保障资金163.7亿元、低保资金26.3亿元、五保供养资金3.8亿元、医疗救助金4亿元、救灾资金3.2亿元、困难职工帮扶资金2833万元。3000多户超期安置对象得到妥善安置。新建和改扩建敬老院86所、“村级五保之家”26所，五保集中供养率由7.5%提高到13%。市本级养老公寓建设顺利推进。市殡仪馆、大山岭陵园和烈士纪念园建成使用。建成廉租住房2.7万套、135.7万平方米，改造城市和独立工矿棚户区148.2万平方米，发放廉租住房补贴1.5亿元，受益人口13.8万户、40万人。累计发放住房公积金贷款40亿元，为9万户职工家庭解决了住房困难。为城乡低保户、五保户发放价格临时补贴3728万元。建成各类农村饮水工程893处，解决147.6万农村人口饮水安全问题。4县84个乡镇建成山洪灾害预警预报系统，治理地质灾害隐患点和矿山环境破坏区36个。减少贫困人口20万人。民族地区、高寒山区脱贫解困工作全面启动。2个单位被国务院授予全国民族团结进步模范集体。

  6、民主法治建设得到新加强。这五年是我市行政效能最高、发展环境最优、和谐社会建设成效最好的时期之一。五年共办结人大代表议案和建议1070件、政协提案1874件，办结率100%。大力实施政务公开和政府信息公开，电子政务和电子监察覆盖全市，隆回被列为全国政务公开和政务服务试点县。市本级行政许可事项减少78项，办理时限缩短50%以上。开展作风建设年活动，机关行政效能不断提升。“法治邵阳”创建活动成效明显，“五五”普法顺利完成。审计监督职能加强，行政监察力度加大。安全生产形势总体向好，事故总量、伤亡人数逐年下降。创新社会管理，全面推广群众工作站，积案化解有效推进，信访渠道畅通有序。严厉打击违法犯罪，破获了一批影响大的危害国家安全案件和刑事案件，社会政治大局和谐稳定。国防动员工作和国防后备力量建设进一步加强，驻邵人民解放军和武警部队在支持和参与地方经济建设、抗灾救灾以及维护社会稳定中作出了重要贡献。

  五年来，统计、人事、编制、海事、档案、地震、人防、外事、侨务、贸促、老龄、文联、残联、宗教、工会、共青团、妇女儿童、台湾事务、地方志、工商联、社会科学、红十字会、无线电管理、驻外办事机构等部门单位做了大量工作，取得了良好成绩。国家安全、金融、工商、邮政、通讯、供电、气象、水文、石油、烟草、盐务、铁路、技术监督、食品药品监督、高等院校、科研院所等中央和省驻邵各单位为全市经济社会发展提供了有力支持。

  各位代表，2012年是本届政府任期的最后一年。这一年是我市推进工作力度最大、出台政策文件频次最多、发展亮点纷呈的一年。一年来，我们高举加快发展旗帜，坚持稳中快进，圆满完成了市十四届人大五次会议确定的各项目标任务。我们着重完善思路，发展目标更加明确。市委、市政府组织开展了“加快邵阳发展大家谈”和心态教育活动，凝聚全市人民的智慧和力量，出台了《加快邵阳发展的决定》等纲领性文件，提出了把邵阳建成“世界著名的旅游胜地、享誉中外的文化名城、国家重要的交通枢纽、湘桂边陲的物流中心、产业兴旺的经济强市、我省西南的生态屏障、民主法治的和谐社会、全面小康的幸福家园”的宏伟目标，形成了加快发展的共识，增强了加快发展的信心，努力实现“三个高于”，即所有经济指标增速绝对高于我省中西部地区增速、主要经济指标增速明显高于我市去年增速、重大经济指标增速适当高于全省各市州增速，保持了经济较快增长。我们围绕市区“四个十大工程”，强力推进179个重点项目建设，总投资超过1380亿元，为历年最高，一批重点项目陆续开工，一批重大工程顺利完成。社会投资始终保持高位运行，投资对经济增长的贡献率保持在75%以上。我们强力推进市容秩序、环境卫生、交通秩序、违法建设、渣土运输、户外广告、路灯亮化、园林绿化八大专项整治行动，引进专业公司从事清扫保洁和园林绿化工作。财政投入2亿元，全面改造市区340条小街小巷。中心城区的环境卫生和交通秩序发生较大变化，得到广大市民一致好评。我们以绩效考核为抓手，积极为民办实事、解难事、做好事，21项为民办实事指标全面完成或超额完成。新型农村养老保险实现全覆盖，102万60岁以上农村老人按月领取养老金。2.5万老工伤人员纳入工伤保险统筹范围。开工建设6392套廉租房、2981套公租房，改造农村危房21956户。社会事业同步跟进，公共服务能力大幅提高。

  各位代表，上述成绩的取得，得益于全市810万各族人民的团结奋斗，得益于历届市委、市政府打下的良好基础，得益于市委的坚强领导，得益于市人大、市政协的监督、协商和支持，得益于邵阳军分区的真诚帮助。在此，我谨代表市人民政府，向作出巨大奉献的全市人民表示崇高的敬意！向给予政府工作大力支持的人大代表、政协委员和离退休老同志，向各民主党派、工商联和各界人士，向中央、省驻邵单位和驻邵人民解放军、武警官兵，向所有关心、支持和参与邵阳建设的同志们、朋友们表示由衷的感谢！

  二、今后五年的目标任务

  各位代表，五年的成绩来之不易，我们在充分肯定成绩的同时也清醒地看到，要全面建成小康社会，我们还面临着很多困难和问题。全球经济形势存在许多不确定性因素，我国经济进入低速发展时期，我市经济社会发展处在中等收入偏低的欠发达状况。主要表现在：经济总量太小，与810万人口的大市不相称；人均占有量太少，与实现小康社会目标差距太大；经济结构不优，一产业在三次产业中的比重过高，二三产业比重偏低；经济运行质量不高，财政收入占GDP比重不高，非税收入占财政收入比重过高；城镇化水平较低，城镇化率比全省低10.5个百分点；城乡居民增收困难，政府服务意识不强，发展环境不优，行政不作为、乱作为现象时有发生等。

  各位代表，看到困难和问题，增强了我们加快发展的紧迫感和危机感，与此同时，我们也看到加快发展充满了极大机遇。未来五年是全面建成小康邵阳的决定性阶段，更是我市加快发展的重要战略机遇期。我国国际地位、国际影响力和综合国力的显著提升，必将为我市加快发展提供难得契机。我市近年经济社会长足发展，人均GDP超过2000美元，已进入快速发展时期，在全面建成小康社会过程中，我们创造和具备了后发赶超的新优势。目前，全市或全部或部分享有国家贫困县、西部地区开发、中部崛起、武陵山片区区域发展与集中连片扶贫攻坚、革命老区、少数民族县、湘西地区开发、问题型老工业基地调整改造、衡邵干旱走廊综合治理和省贫困县等优惠政策，政策优势显现，政策机遇难得。沪昆高铁、怀邵衡铁路、武冈机场、超薄超强平板显示玻璃、衡邵干旱走廊综合治理、娄邵盆地高标准基本农田建设等重大项目将陆续开工和竣工，为邵阳区位条件、产业发展和基础设施的根本性改变打下坚实基础。市委十届三次全会提出了“八个建成”的宏伟目标，为我们指明了发展方向。全市上下加快发展的共识已经形成，加快发展的信心更加坚定，加快发展的热情空前高涨。特别是党的十八大开启了全面建成小康社会的伟大进程，必将为我市经济又好又快发展注入强劲动力。可以说，加快邵阳发展其势已成、其时已至。因此，我们将增强信心，振奋精神，科学把握，沉着应对，既敢于直面困难、勇于接受挑战，更善于抓住机遇、大胆开拓进取，赢得发展的主动权，赢得发展的新优势，努力推动邵阳更高水平、更好质量、更长时期的快速发展。

  今后五年工作的指导思想是：全面贯彻落实党的十八大精神，坚持以邓小平理论、“三个代表”重要思想、科学发展观为指导，按照“四化两型”战略部署，围绕“八个建成”宏伟目标，继续坚持“稳中快进”总基调，在加快发展中转方式，在转方式中加快扩大经济总量，持续推动转型发展、开放发展、绿色发展、和谐发展，努力建设经济繁荣、人民富足、生态优良的幸福美好邵阳。

  今后五年工作的奋斗目标是：到2017年底，实现“五个突破”和“五个翻番”：地区生产总值年均增长12%，突破2000亿元，实现总量翻番；财政收入年均增长20%，突破200亿元，实现总量翻番，提高财政收入占GDP、税收收入占财政收入的“两个比重”；规模工业增加值年均增长17%，达到810亿元，实现总量翻番；固定资产投资年均增长30%，达到2850亿元，实现总量翻番；社会消费品零售总额年均增长16%，达到800亿元，实现总量翻番；城镇居民人均可支配收入年均增长12%，突破25000元；农村居民人均纯收入年均增长12%，突破9000元；城镇化率突破50%。城镇登记失业率控制在4.5%以内。森林覆盖率达到60%以上。万元生产总值能耗、主要污染物排放控制在上级下达的目标以内。做大做强县域经济，争取有4-6个县市GDP过200亿元，财政收入10亿元以上。

  1、坚持产业立市，壮大实体经济。加快产业发展，壮大实体经济，努力形成更加合理的三次产业结构，一产业比重降低到20%以内，二产业比重提高到45%以上，三产业比重保持在35%以上，注重提高经济发展的质量和效益。始终坚持兴工强市战略，把发展工业作为加快发展的第一推动力，到2017年基本形成以百亿元企业为龙头、五十亿元企业为重点、十亿元企业为骨干、亿元企业为群体的“1511”新型工业发展体系。扶持传统产业发展，实现产值达300亿元产业2个，新增产值过百亿元产业3个以上。培育战略性新兴产业，实现产值600亿元以上，高新技术产业产值年均增长25%。建成2个以上年工业总产值超500亿元的工业园区。理顺邵阳经济开发区管理体制，宝庆科技工业园获批国家级经济技术开发区。在确保粮播面积870万亩、粮食总产340万吨的基础上，推进农业产业化和企业化经营，农产品加工规模企业发展到360家，其中年产值1亿元以上的20家、10亿元以上的5家。争创地理标志性产品10个以上。把发展第三产业摆在更加突出位置，坚持文化、物流、旅游三轮驱动，努力培育文化支柱产业，打造湘桂边陲的物流中心，加快建成世界著名的旅游胜地和全国知名的旅游目的地。到2017年，文化产业产值达到150亿元；实现独立上市公司3家，建成年交易额10亿元以上的物流市场15个以上；旅游接待达到3100万人次，实现旅游总收入340亿元，旅游接待人次和旅游总收入年均增长25%以上。进一步理顺市、县（市、区）体制，促进县域经济发展。支持邵东率先发展，重返全省十强。支持武冈建成湘西南中心城市。立足民营经济发展和特色产业优势，形成各县市区错位发展和竞相崛起的生动局面。

  2、坚持城乡统筹，扩大城镇规模。着力挖掘城镇化在扩大内需中的最大潜力，努力实现城镇化与进城农民市民化齐头并进。基本建成以市区为核心、县城为依托、重点城镇为支撑、一般乡镇为基础的网络型城镇体系。东部以中心城区为主城，新邵、邵东为辅城，邵阳、隆回为卫星城，加快中心城区与邵东、新邵、邵阳、隆回县城的融城步伐。基本建好东部城镇群的城际快速干道和环路路网，形成半小时经济圈。抓紧中心城市规划修编，实现中心城区面积80平方公里、人口80万。按照“双百”山水园林城市定位，以三大环城路网建设为核心，不断拓展发展空间。三年建成邵阳大道新城区。抓好管网、公交、停车场等城市基础设施配套，加快自来水扩容提质改造，推广节水型社会建设。推进城市网格化管理，建立完善城市管理长效机制。尽快建成国家卫生城市。西部以武冈为中心，新宁、洞口、绥宁、城步为节点，加强生态建设，建成崀山世界地质公园，打造旅游经济圈。支持乡镇建设，推进“百城千镇万村”新农村建设工程，充分发挥小城镇聚集产业、吸纳人口的作用，新增一批全国全省的示范镇、中心镇和特色镇，提高城镇化率。五年建好50个新农村示范镇、100个示范片、1000个示范村。加强生态文明建设，开展“三区三边三年”绿色行动，建成国家森林城市。持续推进退耕还林、石漠化治理工程，促进生态自然修复，保护良好生态环境，建设天蓝、地绿、水净的美丽邵阳。落实最严格的耕地保护制度，加大农村土地综合整治力度，规范农村土地流转经营，推广农村集中成片建房模式。强化能评监管，推进公共机构和行业节能。抓好污染隐患整治，促进城乡环境逐步好转，确保城乡居民对环境的满意率达到75%以上。

  3、坚持投资拉动和项目支撑，完善基础设施。千方百计上项目、增投入、促发展，形成以项目作支撑、以项目为载体、以项目促发展的良好局面。结合上级政策要求和邵阳发展实际，规划包装好总投资1.2万亿元的1300个重点项目，争取760个规划项目进入省级预选规划，力争未来五年项目投资占全省10%以上。建成怀邵衡铁路，争取建设邵冷、凯靖永郴、常桂铁路和长株潭到邵阳城际铁路，推进“三纵三横一连”网络建设。建成武冈至靖州、邵阳县白仓至新宁、陕西安康至广西北海邵阳段、邵阳至隆回金石桥、洞口至怀化溆浦、新宁至永州等高速公路，构建“三纵三横两连”体系。完成1000公里国省干线公路改造任务，连通3000公里的农村公路“断头路”。建好邵阳火车南站综合客运枢纽站和邵坪高铁配套客运站，搬迁邵阳汽车东站、汽车南站。新建新宁、城步到桂林、龙胜等连接高速，打通我市通往广西通道，畅通对接国家北部湾经济区和东盟经济圈的出海出境大通道。建成武冈机场并通航，加快邵东军民两用机场建设。完成衡邵干旱走廊综合治理大部分项目，全面完成娄邵盆地高标准基本农田项目。加快资江干流支流和40条中小河流重点河段治理，全面完成规划内病险水库除险加固任务，完成犬木塘水库和市区第二水源工程建设。按照50年一遇标准建好中心城区防洪堤，加快县城防洪工程建设。建成500千伏变电站1个、220千伏变电站14个、110千伏变电站74个，实现县县拥有坚强电网。完成未改村的农网改造任务。

  4、坚持改革开放，激发经济活力。通过推进经济领域各项改革，提高对外开放水平，使经济发展动力持续增强，经济发展活力充分释放。以纳入全国问题型老工业基地调整改造范围为契机，全面完成国企改制“三年攻坚行动”任务。抓好政府机构改革，逐步理顺行政管理体制。加强投融资体制改革，组建交通投资等融资平台，增强融资能力。推进农业经营体制改革，稳妥推进农村土地经营权流转，实施征地制度改革。加快文化体制改革，建立适应市场需求与文化发展方向的体制机制。积极对内开放，坚持产业招商，引进先进装备制造、新能源、电子信息、文化旅游、商贸服务等产业的战略投资者。突出金融等生产性服务业招商，争取商业银行到我市设立分支机构。加强与央企、省企、上市公司对接，引进一批世界500强、中国500强企业落户邵阳，积极发展总部经济。继续做好引老乡回故乡建家乡工作，每两年召开一次邵商大会。激发民资投资热情，引进和扩大民营资本对社会事业和民生工程的投入。抓好本地煤炭企业兼并重组。扩大对外贸易，努力拓展欧盟、北美、中东等国外新兴市场，鼓励和支持我市企业承包国际工程，拉动外贸出口和劳务输出。力争5年内培育年进出口总额上亿美元的企业5-6家、过5000万美元的企业8-10家、过3000万美元的企业20家，外贸进出口总额超过12亿美元。设立邵阳海关、出入境检验检疫局和公路口岸。

  5、坚持社会事业快速发展，完善基本公共服务体系。在巩固普及九年义务教育的基础上，发展学前教育，新建幼儿园300所、义务教育合格学校1203所，普及高中阶段教育，合并组建幼儿师范高等专科学校，整合职业技术学校创办职业技术学院，形成惠及全民的公平教育，初步建成教育强市。加大农村学校和城镇薄弱学校投入力度，提高义务教育阶段贫困寄宿生资助比例，确保特困家庭学生、孤儿和残疾少年儿童免费入学、免费食宿。调整和扩大市城区小学、中学规模和布局，基本解决大班额问题。加快学校教育信息化发展，推行“班班通”工程。围绕建成享誉中外的文化名城，加强文物发掘和保护，传承文化遗产，逐步恢复“宝庆八景”，支持绥宁侗族村寨申报世界文化遗产。建好市本级和各县市文化艺术中心、博物馆、科技馆，抓好图书馆、文化馆、美术馆以及乡镇综合文化站、群众文化广场和村（社区）文化活动中心建设，并免费向群众开放。实现广播电视由“村村通”向“户户通”延伸。加快以中国君子文化产业园为重点的文化产业发展。继续推进公共卫生服务均等化、医疗资源配置合理化、城乡卫生发展一体化。重点抓好国家和省扶持的县级医院、中心乡镇卫生院、县级疾病卫生防治机构的项目建设，加快乡镇卫生院和市区社区卫生服务机构达标步伐，建成市中心医院新院。全市新增病床位5000张以上。抓好重大疾病防控，提高群众健康水平。发展体育事业和体育产业，建成市体育中心，申办2018年第十三届省运会。坚持计划生育基本国策，着力增进计生家庭福祉，统筹解决人口问题，促进人口长期均衡发展，实现人口素质明显提高，人口结构明显改善。加快产学研合作，不断提高科技创新能力，不断提高科技对经济发展的贡献率。支持国防建设，走军民融合式发展道路。

  6、坚持民生优先，提高群众生活水平。始终把解决民生问题放在政府工作的首位，把更多的财力投向民生领域，多上快上关系民生的项目，让群众生活越过越好。完善市场化的就业机制和对困难就业群体的帮扶机制，未来五年新增城镇就业20万人、农村劳动力转移就业27万人，城镇登记失业率控制在4.5%以内。坚持全覆盖、保基本、多层次、可持续，强化社会保险扩面征缴，逐步建立城乡低保标准自然调整机制，实现全员覆盖目标。提高五保供养和优抚保障水平。大力推进农村敬老院改扩建进程，基本做到“一乡一院”，五保集中供养率达到25%。逐步完善社会救助体系，大力发展慈善事业。高度关注下岗职工、退休人员以及失地农民等群体的生活，让他们得到更多实惠。扎实建好农村危房改造工程。强力推进住房公积金制度向非公有制企业和进城劳务人员延伸，新归集住房公积金50亿元、发放住房贷款65亿元以上。全面推进保障房建设，积极帮助中低收入家庭解决住房问题。突出基础设施、生态环境、特色产业建设，落实好武陵山片区区域发展与集中连片扶贫攻坚政策和项目，帮助50万贫困人口脱贫致富。加大扶持力度，促进民族地区和高寒山区发展。依法保护群众正当权益，努力解决群众合理诉求。加强和创新社会管理，增强城乡社区服务功能，充分发挥群众参与社会管理的基础作用，全面推进社会稳定风险评估，有效化解各类矛盾。严厉打击各类违法犯罪，维护社会治安稳定。加强安全生产和食品药品监管，努力遏制安全事故发生，确保人民群众生产安全、生活安全和社会公共安全。

  7、坚持清正廉明，打造服务型政府。能否实现今后五年的奋斗目标，政府的作为是关键。我们将按照“干部清正、政府清廉、政治清明”的总体要求，逐步建立职能科学、结构优化、廉洁高效、人民满意的服务型政府。强化法制意识，各级政府及其工作人员要学会运用法治思维和法治方式处理改革、发展、稳定中的各种矛盾和问题，依法接受人大及其常委会的法律监督、工作监督，坚决执行人大的决议决定，自觉接受政协的民主监督，认真办理人大代表建议议案和政协提案。认真落实《法治湖南实施纲要》，完成“六五”普法任务。进一步规范行政执法行为，做到规范执法、公正执法、文明执法。强化职能意识，政府必须在法律法规授权的范围内行使职权，凡是法律法规没有授予的权力，政府就不得行使，解决乱作为的问题；理顺政府与市场的关系，凡是通过市场能做到的事，政府就要放手，解决大包大揽的问题。强化公开公平意识，加大政务公开力度，加强电子政务建设，提高权力运行的透明度。强化人民主体意识，政府全体工作人员要把人民放在心中最高位置，所做一切事情都要以群众满意为出发点和落脚点，多做知民情、解民忧、暖民心的好事实事。强化效能意识，深入开展机关效能和作风建设，严格政府绩效管理，促进政府工作全面提速提质。强化诚信意识，提高政府公信力，坚决防止失信于民的言行，通过政府诚信，带动商务诚信、社会诚信。强化廉政意识，坚持廉洁从政，坚持用制度管权、管事、管人。充分发挥监察、审计的监督作用，规范项目招投标、国有土地出让、产权交易、政府采购、公务消费等行为，特别加强重大项目、重点领域的审查和监管。积极推进节约型机关建设，勤俭办一切事业。

  三、新一届政府开局之年的重点工作

  2013年是新一届政府任期的第一年，做好明年的政府工作十分关键。主要预期目标是：地区生产总值增长12%，规模工业增加值增长17%，固定资产投资增长30%，社会消费品零售总额增长16%，财政收入增长20%，城镇居民人均可支配收入增长12%，农村居民人均纯收入增长12%，居民消费价格涨幅控制在4%以内，人口自然增长率控制在7.5‰以内，万元生产总值能耗下降3%，主要污染物排放总量控制在省政府下达的目标之内。

  重点抓好十一个方面的工作：

  1、狠抓工业不放松。把壮大工业作为加快发展的主战场，领导精力、各类资金和生产要素向工业集中。做大做强产业集群。新增产值过200亿元产业2个、过100亿元产业1个。加快骨干企业发展。重点实施十大工程，即三一湖汽完成整体搬迁并建成重卡生产线，湘窖酒业建成2万吨清香型白酒生产车间，宝庆煤电二期完成立项审批并启动工程前期建设，九兴鞋业一期600万双耐克鞋生产线投产，超薄超强平板显示玻璃项目完成一期主体工程，宝兴科肥年产15万吨硝酸铵项目建成投产，大成科技国家农村移动电子商务示范项目完成省内平台建设并开始全国试点，百威啤酒年产50万吨啤酒生产线完成主体工程，二纺机新址二号厂房建成投产，湘中制药完成一期主体工程和设备安装调试。同时，中小微企业创业基地和邵东皮具基地初具规模，发制品暨外贸转型升级专业型示范出口基地、湘中铸造中心、恒远资江水电大中型水轮发电机组生产线开工建设。每个县市区重点扶持2-3个工业项目。加快企业改制。打好企业改制攻坚战，市属36户国有工交企业改制完成70%以上，30户国有流通企业和36户集体企业完成改制40%以上。强力推进各县市企业改制工作。抓好园区建设。在全市形成“抓工业必须抓园区”的广泛共识，突出抓好园区产业集聚，引导企业入园发展，新增入园企业60家以上，省级园区规模工业增加值增长20%以上。宝庆科技工业园按照调整园区规划、理顺管理体制、建立融资平台、加快基础配套、引进战略投资伙伴的思路，强力推进园区建设。启动再生资源、生物医药、特种造纸、发制品加工、农产品加工、中小企业产业园等特色园区建设。

  2、围绕农民增收加快农业产业化发展。稳定粮食生产。优化财政投入，强化行政推动，努力“扩双压单”，着力恢复双季稻，大力发展玉米等旱粮生产，确保粮食总播面积稳定在870万亩以上，总产340万吨以上。经济作物总面积稳定在240万亩以上。实现烟叶生产30万担目标。推进农业产业化。推行“公司+基地+农户”等模式，积极发展生猪、乳业、橘橙、药材、油茶、楠竹、烟叶、金银花等特色产业，培育一批农产品精深加工骨干企业，做大做强龙头企业，新增省级龙头企业5家。加强农村机耕道建设，推广农机项目，搞好农机化专业服务，提高农业机械化水平。拓宽农民增收渠道。扩大杂交水稻制种的范围和面积，提高农民种粮收入。加快发展农村二、三产业，提高农民非农产业收入和劳务性工资收入。实施“阳光工程”、“温暖工程”、“雨露计划”和农民创业培训，扩大农民就业，增强农民创业能力，实现农村劳动力有序转移。推进农业经营体制改革，引导农企合作，发展壮大农民专业合作组织，新增农民专业合作组织100个以上。全面落实惠农减负政策，严格涉农收费管理，切实减轻农民负担。加强连片综合开发。按照“办点示范、连片推进、逐步扩面、整体提高”的思路，整合水利建设、综合开发、环境整治等各种涉农资金，打造新农村示范镇12个、示范片20个、示范村200个。投入农业综合开发资金8亿元以上，建设土地治理项目区13个，实施产业化经营项目28个。扶持民族地区、高寒山区发展，帮助贫困地区改善基本生产生活条件。加快质量标准化体系建设，抓好农产品生产技术标准的制定、宣传、推广与实施，严格农产品基地准出、市场准入和检验检测，确保放心农产品进入千家万户。加强动物防疫检疫，防止重大动物疫病发生和流行。

  3、加快服务业发展，提高第三产业比重。壮大旅游业。完成全市旅游产业发展总体规划修编和重点景区建设性详规编制，制定邵阳西部生态文化旅游圈发展规划。启动游客服务中心、蔡锷故居建设。利用崀山世界品牌优势，发挥崀山龙头作用，完善崀山、南山、云山、花瑶、黄桑、罗（请造字：草字头+那）溪、紫薇博览园、莲荷生态公园、白水洞等景区配套设施，抓好崀山创5A级景区和南山、黄桑、武冈古城、紫薇博览园创4A级景区工作，发展休闲农业、休闲林业，支持新宁创建国家级旅游标准试点县、隆回创建省级旅游强县。扶持特色旅游商品开发项目，创建一批省级旅游购物示范点。设立旅游发展基金。牵头举办“2013大桂林原生态民族风情节”。全年接待游客1900万人次，旅游总收入达到140亿元以上。繁荣市场消费。继续加大“万村千乡”工程推进力度，新建改造配送中心2-3家、农家店300家。加强物流基础设施建设，以农产品加工配送中心为重点，新建和改造一批现代化的大型批发市场、农贸市场和蔬菜市场，制定全国农产品现代流通综合试点城市规划，推进农产品现代流通体系建设。推进商贸流通、餐饮食宿、娱乐休闲、医疗保健等服务业升级改造，引进友谊阿波罗、沃尔玛等大型购物超市。加强建材、汽车、服装等专业市场建设，打造一批专业街区和品牌名店。加快生活性服务业发展，培育壮大养老、保健、娱乐、体育等新兴业态。发展金融等生产性服务业。加快发展生产性服务业，提高生产要素的保障能力。加大政府支持力度，搞活金融服务业。研究出台加快金融发展的若干政策性意见，规划建设金融集聚区，加快组建邵阳农商行，引进1-3家股份制银行或其他金融机构，推进农村中小金融机构建设，启动金融生态模范城市创建工作。发展小额贷款公司，扶持现有融资性担保公司做大做强。推进本土重点企业上市，实现零的突破。促进保险市场健康发展，实现保费收入增长10%以上。利用我市区位交通优势，积极鼓励民间资本投资生产性物流中心建设，扩大生产性物流业的规模和数量。邵阳学院和各类工程技术学院、学校要面向工农业生产提供高技术、高技工服务，鼓励市内外高校在我市建立各类科学技术的信息、咨询等中介服务平台。

  4、继续强力推进市中心城区的提质扩容。完成《邵阳市城市总体规划（2011-2030）》、东部城镇群城镇体系规划和420平方公里城乡统筹先导区建设规划，修改中心城区土地利用总体规划，加快编制火车北站片区和洋溪桥片区控制性详细规划。制定并完善工业园区、资江两岸景观规划、户外广告专项规划以及邵水东路、邵水西路南段景观工程设计。坚决制止和纠正规划实施中的随意性，切实维护规划的严肃性和权威性，抓好拆违控违工作。组建交投和园区等融资平台，发挥城投、经投、国资投的作用，多渠道筹措城市建设资金，全年投入城建资金50亿元以上。重点构建中心城区快速环线和东部城镇群快速通道，建设城市桥梁，打通城区瓶颈路。启动邵坪高速及其城区连接线建设，完成东互通大道主路面工程。启动邵阳大道东延至邵东道路建设，开工建设学院路延伸段，实施蔡锷路提标，加快市区同邵东、邵阳、新邵三县的融城步伐。继续加快推进十大城建工程和十大民生工程的建设，加固维修邵水桥，加快建设雪峰桥、桂花桥、云山大桥。完成大祥立交桥西向、邵水东路六中段、邵水西路延伸段，启动新华路北段隧道建设。推进城市主次干道和小街小巷提标改造，完成西湖路、宝庆路、红旗路、敏州西路、城北路西段、建设中路、双坡南路、五一南路、东大路二期、铁砂岭路及340条小街小巷提质改造，启动雪峰南路延伸段、建设南路延伸至蔡锷故居并连接至贺绿汀故居道路、雪峰北路、邵檀路建设。完成西苑公园一期工程、紫薇博览园二期工程、六岭公园建设及人民广场改造，启动北塔公园、爱莲池公园建设，实施资江、邵水、红旗渠风光带建设。打造东互通大道、邵阳大道、学院路、西湖路、蔡锷路、宝庆路等园林景观带。抓好园林绿化，绿地率、绿化覆盖率分别达到31%、35%以上，人均公共绿地面积7平方米以上，建成省级园林城市。完善公共基础设施配套，全面铺开城镇污水管网建设，加快江北污水处理厂一期、洋溪桥污水处理厂配套管网改扩建一期和市污水处置中心建设，继续抓好资江、邵水中心城区段清污。提高城镇燃气气化率，规划建设市区到邵东、邵阳、隆回三县的天然气输送管道。加快人防新型疏散基地和人防培训中心建设。新建改建旱厕100个、垃圾中转站20个、农贸市场3个，增设果皮箱、垃圾桶3000个和垃圾围300个，启动江北蔡锷路和桃花新城公交停车场建设，建成机动车驾驶员培训考试中心。加强小街小巷灯化美化，规范设置主干道路两旁门店招牌和灯饰广告。市行政中心、湘林国际大酒店主体工程完工。抓好城区征地拆迁，推进安置方式改革，加快10处安置点和保障房建设。建设数字邵阳地理空间框架平台，建成市区数字城管平台。理顺城管体制，强化三区城管职责，构筑城乡一体、层次分明、条块结合、重心下移的管理格局。组建专业化垃圾运输公司，规范渣土密闭运输管理，落实建筑工地净车出场制度。继续开展城市管理综合整治，实施“限摩限电”，推动园林绿化、市容环卫、市政设施管护制度化、常态化，力争中心城区面貌再有一个大的改变，确保顺利通过省级卫生城市复查，进入创建国家卫生城市申报程序。

  5、努力改善城乡基础设施。抓住国家扩大投资机遇，争取省委、省政府出台支持我市加快发展的专门文件，实施一批事关我市全局和长远的重点基础设施工程。围绕十大交通工程，怀邵衡铁路实质性开工，加快娄邵铁路扩改和沪昆高铁建设步伐。确保洞新、邵安和怀通高速邵阳段建成通车，实现全市12个县市区通高速公路。邵坪高速公路全线实质性建设，开工建设武冈至靖州高速公路，争取邵阳县白仓至新宁高速公路正式动工。推进邵塘一级公路等在建7条266公里的国省干线建设，确保建成3条138公里，新开工9条314公里。完成500公里的农村“断头路”连通。建成3个县级客运站。动工建设武冈机场，积极推进邵东机场前期工作。完成133座小二型水库除险加固，启动下源、洛口山、马皇冲三座中型水库整治工程，竣工24个中小河流治理项目。建设农村饮水安全工程，解决30万农村人口饮水安全问题，加强饮水工程后续管理。争取衡邵干旱走廊综合治理项目获批，力争犬木塘水库审批立项，开工建设东风水库及灌区工程，启动市区第二水源建设项目。抓好土地整理，新增一批高标准农田。全面完成列入国家计划的9个县市山洪灾害防治县级非工程措施项目建设。新建220千伏变电站2个、110千伏变电站4个。支持宝庆电厂二期、南山风电二期、风雨殿风电场、生物质发电和太阳能等新能源项目建设。完成大圳供区改制并纳入大电网代管，启动电网改造。

  6、努力形成全民招商引资热潮。把招商引资作为加快发展的“出路工程”。制定出台招商引资优惠政策，整合招商资源和力量，强化市、县、乡三级政府及其部门招商引资的责任，带动全民招商，营造亲商、重商、爱商、富商的浓厚氛围，打造吸引客商的“政策高地”和“投资洼地”。立足产业招商、网上招商、以商招商，主动承接沿海产业转移，积极引进战略投资者。每个县市区引进产值过亿元工业企业1家以上。提高2012邵商大会签约项目履约率，力促履约项目尽快落地建成。全年利用外资增长10%，内联引资增长20%以上。开工建设邵阳海关、出入境检验检疫局大楼。积极开展申报工作，获批全国承接产业转移示范基地。扩建境外联络处，加强与国外商协会的交流与合作。加快建设鞋业、打火机、发制品、皮草皮革、农副产品、医化产品、箱包服装、五金机械、竹木制品、水电设备十大外贸出口基地，引导九兴鞋业等出口企业自营出口。外贸进出口增长20%，新增境外投资、新签国际工程合同额、外派劳务指标增长15%。

  7、推进生态文明建设。我们将更加自觉地尊重自然、顺应自然、保护自然，力争全市或大部分县市进入全国主体功能区。强力推进绿色邵阳建设，强化三区主体责任，持续开展“三区三边三年”绿色行动，完成各类造林35万亩。全面深化石漠化综合治理和退耕还林成果巩固工程，启动市级生态公益林补偿机制。全市森林覆盖率达到58.5%，其中市区森林覆盖率达到28%以上。扩大森林、湖泊、湿地面积，建成1-2个湿地公园。增强城乡防洪抗旱排涝能力，加强防灾减灾体系建设，提高气象、地质、地震灾害防御能力。切实抓好以生态县、环境优美乡（镇）、生态示范村为载体的生态建设与保护工作。巩固西部县市的生态优势，大力实施楠竹低改和特色资源培育。加大煤、铁、锑、金等矿产资源的勘查力度，整顿和规范矿产资源开发秩序，推进绿色矿山建设，恢复治理矿山环境。在农村开展以畜禽养殖污染为重点的环境整治，逐步改善村容环境卫生。推广清洁能源，新建户用沼气池3500口、养殖小区和联户沼气工程279处、大中型沼气工程4处，推广太阳能利用器具2000台。加快建设“十大环保工程”和节能减排重点工程。开展云峰水泥、宝兴科肥循环经济试点，在建材、化工、冶炼等高耗能行业启动一批循环经济和资源综合利用项目。加强环境监测，强化环评、能评设置，推广节能、节地、节水、节材，全面完成年度各项刚性考核指标。按照产业政策和市场准入标准审批项目，严格控制主要污染物排放新增量。抓好减排措施的落实，按计划推进污染减排工程项目。

  8、加快社会事业发展。加大投入力度，优化资源配置，着力提高社会管理和公共服务水平。加快发展文体事业。修订完善《邵阳市文化产业“十二五”发展规划》，挖掘整理文化产业资源，推进市文化艺术中心、非物质文化遗产保护基地等重点文化项目建设，启动中国君子文化产业园项目建设，开始第二轮市志总篡工作。实施文化惠民工程，抓好“三馆一站”免费开放，继续做好“送戏下乡”和公益电影放映工作。建成市广播电视监控中心。加强文化市场综合执法监管，建成网络文化、新闻出版、娱乐文化等监管平台，深入开展“扫黄打非”专项整治行动。建设市体育中心，筹备申办省运会。办人民满意教育。紧紧围绕建成教育强市目标，加快教育改革和发展步伐。加速示范性幼儿园与乡镇中心幼儿园建设。邵阳学院和邵阳医学高等专科学校合并组建湖南工程科技学院。推进邵阳幼儿师范高等专科学校合并组建工作。建成市区祥凤学校。均衡发展城乡教育，加大义务教育合格学校建设力度，完成义务教育合格学校和农村公办幼儿园的年度建设任务，保障好进城务工人员子女平等接受教育的权利。努力改善医卫条件。进一步加强医疗保障、医疗服务、公共卫生、药品供应及监管体制、运行机制建设，强化全民基本医保、基本药物制度。抓紧市中心医院新院建设。完成一人民医院儿科病房主体工程。基本建成市应急备灾指挥中心项目。落实好市本级和12个县市区卫生监督所、80所乡镇卫生院和8所农村卫生救援中心建设任务。提高科技创新能力。加快科技型企业和战略性新兴产业培育，推进科技型中小微企业创新发展，新增高新技术企业5家以上。实现专利申请1100件，授权650件。做好人口计生工作。全面完成国家免费孕前优生健康检查任务，出生人口性别比有所下降，新增1个以上县市区为全国计划生育优质服务单位。同时，认真搞好第三次全国经济普查，全面掌握我市经济规模和经济结构。

  9、优先保障和改善民生。努力实现群众收入增长与经济发展的良性互动，满足群众的新期待，增强群众的幸福感。全年新增城镇就业4.6万人，培训农村劳动力7万人，转移农村富余劳动力就业稳定在130万人以上。新增基本养老、医疗、工伤、生育、失业保险人数1.2万人、2.5万人、2万人、0.8万人、1.98万人。提高五保对象供养标准，完成敬老院的改扩建任务。开工建设2-3个老年公寓，推进社会化养老。加强低保资金管理，完善城乡医疗救助制度，健全大病救助机制。规范救灾款物的管理和使用，促进救灾救济工作规范化。新建廉租住房3716套、18.5万平方米；新建公共租赁住房11306套、67.8万平方米；改造城市棚户区7008户、45.5万平方米；发放租赁补贴4万户、4000万元。用好武陵山片区区域发展与集中连片扶贫攻坚政策，加大扶贫工作力度，全年帮助10万贫困人口脱贫致富，确保贫困地区农民人均纯收入增幅高于全市平均水平。

  10、全力确保安全和维护社会稳定。强化安全隐患排查整治，深化“打非治违”专项行动，确保不发生重大以上安全生产事故，确保安全生产事故指标不超省控目标，争创全省安全生产先进单位。切实抓好防汛抗旱工作，严密防范山洪地质灾害。推进社会管理创新，逐步完善社会管理格局。加强社会管理综合治理，深入开展“打黑除恶”专项行动和治安重点整治，严厉打击“两抢一盗”等各类违法犯罪活动，全力抓好禁毒整治工作，加强和改进动态化信息化条件下公安基层基础工作。加大“六五”普法力度，全面推进法治邵阳建设。进一步深化群众工作站工作，畅通信访渠道，有效预防和及时化解社会矛盾，切实维护好企业和群众的合法权益。

  11、努力提高政府执行力。提高执行力就是提高落实的能力，解决落实能力不强的问题。政府所有工作都要在党委的统一意志下开展，政府必须保证政令畅通，决不允许“上有政策、下有对策”，决不允许有令不行、有禁不止。政府各部门、市与各县市区要精诚团结，合作共事，决不允许推诿扯皮、互不买账。充分发挥村委会、乡镇政府、社区和街道办事处以及各基层单位作用，强化基层组织的责任，实行向基层倾斜的各种政策，尤其要建立逐年稳定增加的经费保障机制。从严治政，强化行政问责。政府各项工作都要落实到单位到人，实行督查督办制、定期反馈制、办结销号制和责任追究制，力戒议而不决，决而不行，行而不果。

  各位代表，全市人民的重托庄严而神圣，我们肩负的任务光荣而艰巨。空谈误事，实干兴邵。宏伟蓝图，美好愿景，只有靠实干才能变成现实。让我们在中共邵阳市委的坚强领导下，牢记重托，奋发图强，高举加快发展旗帜，再创邵阳历史辉煌，为建设810万邵阳人民全面小康的幸福家园而努力奋斗！
